# Leveraging Vision Foundation Model via PConv-Based Fine-Tuning with Automated Prompter for Defect Segmentation

**DOI:** 10.3390/s25082417

**Published:** 2025-04-11

**Authors:** Yifan Jiang, Jinshui Chen, Jiangang Lu

**Affiliations:** State Key Laboratory of Industrial Control Technology, College of Control Science and Engineering, Zhejiang University, Hangzhou 310027, China; zjujyf@zju.edu.cn (Y.J.); jschen@zju.edu.cn (J.C.)

**Keywords:** defect segmentation, vision foundation model, segment anything model, low-rank adaptation, partial convolution, automated prompter, parameter-efficient fine-tuning

## Abstract

In industrial scenarios, image segmentation is essential for accurately identifying defect regions. Recently, the emergence of foundation models driven by powerful computational resources and large-scale training data has brought about a paradigm shift in deep learning-based image segmentation. The Segment Anything Model (SAM) has shown exceptional performance across various downstream tasks, owing to its vast semantic knowledge and strong generalization capabilities. However, the feature distribution discrepancy, reliance on manually labeled prompts, and limited category information of SAM reduce its scalability in industrial settings. To address these issues, we propose PA-SAM, an industrial defect segmentation framework based on SAM. Firstly, to bridge the gap between SAM’s pre-training data and distinct characteristics of industrial defects, we introduce a parameter-efficient fine-tuning (PEFT) technique incorporating lightweight Multi-Scale Partial Convolution Aggregation (MSPCA) into Low-Rank Adaptation (LoRA), named MSPCA-LoRA, which effectively enhances the image encoder’s sensitivity to prior knowledge biases, while maintaining PEFT efficiency. Furthermore, we present the Image-to-Prompt Embedding Generator (IPEG), which utilizes image embeddings to autonomously create high-quality prompt embeddings for directing mask segmentation, eliminating the limitations of manually provided prompts. Finally, we apply effective refinements to SAM’s mask decoder, transforming SAM into an end-to-end semantic segmentation framework. On two real-world defect segmentation datasets, PA-SAM achieves mean Intersections over Union of 73.87% and 68.30%, as well as mean Dice coefficients of 84.90% and 80.22%, outperforming other state-of-the-art algorithms, further demonstrating its robust generalization and application potential.

## 1. Introduction

As the demand for higher product quality and increased production efficiency in industrial manufacturing has escalated, industrial surface defect detection technologies have garnered considerable attention. The accurate detection and segmentation of surface defects are critical to ensuring product quality, minimizing defect rejection rates, and optimizing manufacturing processes [1]. However, due to the irregularity, diversity, and complexity of industrial surface defects, traditional manual inspection methods are not only time-consuming and labor-intensive but also highly susceptible to subjective biases, making it challenging to achieve high precision and consistency in defect detection [2]. Therefore, automated defect detection using computer vision and deep learning technologies has become a focal point of current research [3,4].

**Figure 1:**
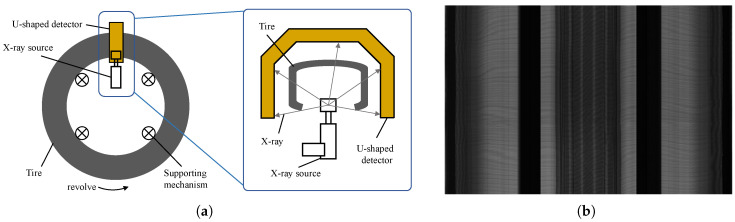
Tire defect detection based on X-ray sensors. (**a**) Diagram of the X-ray imaging system. (**b**) Imaging results captured by the X-ray sensor.

In industrial defect detection, image segmentation techniques are essential for accurately identifying defect regions [5]. In recent years, deep neural network-based segmentation methods, such as FCN [6], U-Net [7], Deeplab [8], and their modified versions, have achieved remarkable outcomes. Most of these segmentation algorithms are preprocessed based on public datasets such as MS COCO [9] and PASCAL Context [10], and then trained on specific datasets. However, collecting industrial datasets is resource-intensive. In taking tire production [11] as an example, detecting defective tires is critical to ensuring product quality and preventing defective products from reaching the market. Machine vision systems are widely employed to capture images and identify defects, with X-ray image sensors commonly used to inspect the internal structure of tires, as shown in Figure 1a. During the image acquisition process, a tire is mounted on an expansion mechanism and rotated 360°. Meanwhile, the X-ray source emits X-rays, which are captured with a U-shaped detector, producing an X-ray image of the tire, as shown in Figure 1b. Each image then requires labeling by experienced personnel. This expert-dependent, costly data preparation process highlights the limitations of the traditional data model’s tightly coupled paradigm.

In addition to conventional deep learning methods, recent advancements in foundation models, such as LLaMA [12] and GPT-4 [13], have sparked a new wave in the field of natural language processing (NLP), presenting new opportunities for the computer vision domain. These large models, by pre-training on vast datasets and leveraging transfer learning with a small amount of labeled data, exhibit remarkable generalization capabilities in downstream tasks [14]. Recent advances in parameter-efficient fine-tuning (PEFT) further enhance the adaptability of vision foundation models [15]. PEFT optimizes the fine-tuning process, reducing computational and storage costs while maintaining high task adaptability. Techniques such as adapters [16], selective parameter adjustments [17], prompt-driven methods [18], and Low-Rank Adaptation (LoRA) [19] have shown significant advantages in improving model adaptability, especially when dealing with tasks where labeled data are scarce and there are large discrepancies in data distributions. The Segment Anything Model (SAM) [20], proposed by Meta AI, is a versatile image segmentation model that has acquired extensive visual knowledge through pre-training on the SA-1B dataset, which includes 11 million high-resolution images and 1.1 billion masks. Different from conventional segmentation models, SAM integrates large-scale datasets with a flexible prompt mechanism, enabling it to segment any object or region in an image, which has demonstrated superior performance in various image segmentation tasks.

The innovative paradigm of SAM, in particular, establishes foundational visual understanding through pre-training on a vast general-purpose dataset and then adapts to specific tasks with a small amount of domain-specific data, demonstrating its potential for industrial applications. However, directly applying SAM to defect segmentation still faces numerous challenges [21]. The first major challenge is that the pre-training data of SAM primarily originate from vast natural image datasets, and differ substantially from industrial surface defect images in terms of feature distribution [22]. Industrial defect images often exhibit specific textures and complex background noise. More specifically, industrial defects such as small cracks, dents, or surface scratches have subtle shapes and texture features, requiring higher resolution and detailed processing of local features [4]. In contrast, natural images generally focus more on global contours and semantic elements. These differences make it challenging for SAM to extract relevant features when leveraging common PEFT methods to process industrial defect images, leading to unstable segmentation performance. Another issue stems from SAM’s reliance on user-supplied visual prompts (e.g., points, bounding boxes, or masks) to generate prompt embeddings, which are cross-attended with image embeddings in the mask decoder to aid in mask prediction. However, industrial applications often demand real-time and efficient processing; thus, manually providing prompts for each image clearly does not meet the needs of automated production environments. Additionally, this prompt-based approach lacks category-specific information, preventing SAM from automatically handling fine-grained defect segmentation tasks [23]. As SAM does support prompt-free input [24], this results in drawbacks in segmentation accuracy and robustness. Finally, SAM generates binary prediction masks based on manual prompts in its standard usage. With a single prompt input, its mask decoder can only differentiate between foreground and background, without linking the segmentation results to specific defect categories. As a result, the segmentation output typically lacks semantic information and cannot be applied directly to industrial contexts where precise defect classification is required.

Building on the research background discussed above, we propose a PConv-based auto-prompting SAM framework for defect segmentation, named PA-SAM, to improve the segmentation performance and application value of SAM in industrial scenarios. Specifically, we first introduce a parameter-efficient fine-tuning (PEFT) method integrating Multi-Scale Partial Convolution Aggregation (MSPCA) into the low-rank linear layers of LoRA. LoRA adjusts the pre-trained features using low-rank matrix transformations, considerably lowering the number of parameters added during fine-tuning while maintaining SAM’s generalization capability. MSPCA, built upon low-rank adjustment, further combines multi-scale convolutions and a partial channel fusion mechanism to better focus on local features. Then, we devise an Image-to-Prompt Embedding Generator (IPEG) to automatically generate visual prompts. This component directly takes the image embeddings as input and sequentially processes them through an Adaptive Multi-Scale Edge Enhancer (AMSEE), Patch Decoder, and prompt encoder, transforming them into high-quality prompt embeddings suitable for predicting segmentation masks. Furthermore, we modified the structure of the mask decoder to output segmentation maps corresponding to the number of categories for the input image, transforming SAM into a multi-class semantic segmentation model.

The main contributions of this paper can be summarized as follows:

- We propose a PEFT method based on partial convolution and LoRA, named MSPCA-LoRA, to facilitate the transfer of knowledge from natural images to industrial defect images. This PEFT method not only ensures extensive knowledge transfer to industrial scenarios but also enhances the model’s sensitivity to local prior knowledge across scales, all while maintaining parameter efficiency.
- We devise the IPEG to automatically generate prompt embeddings, eliminating the need for manual prompt design during both training and inference. These prompt embeddings are then used to guide predicting segmentation masks, thus enhancing SAM’s practical applicability in industrial settings.
- We make slight yet effective architectural adjustments to the mask decoder, transforming SAM into an end-to-end semantic segmentation model suited for defect segmentation tasks. Based on this architecture, we leverage MSPCA-LoRA and the IPEG to build PA-SAM. We conduct extensive comparison and ablation experiments on two common-used defect segmentation datasets, demonstrating the effectiveness of our proposed method for such downstream tasks.

## 2. Related Work

### 2.1. Defect Segmentation Models

With the rapid development of deep learning technologies, remarkable advances have been made in image segmentation [5], particularly in defect segmentation tasks. Defect segmentation is crucial for accurately identifying and localizing abnormal regions within images, and has significant applications in fields such as industrial quality inspection and medical image analysis. Convolutional Neural Networks (CNNs), the foundation of deep learning, are widely used in image segmentation tasks. Early defect segmentation approaches primarily relied on classic CNN architectures, such as the FCN [6] and DeepLab [8] series. As deep learning has progressed, encoder–decoder architectures have evolved, giving rise to more advanced models such as U-Net [7] and SegNet [25]. Yang et al. [26] introduced a Residual Shape Adaptive Dense-Nested U-net, which fuses multi-semantic features using dense skip connections and incorporates a shape-adaptive module for accurate defect localization. Hu et al. [27] proposed an X-ray void image segmentation algorithm based on enhanced DeepLabV3, which leverages MobileNetV2 and AMTPNet to optimize shallow features, leading to notable improvements in improving solder joint void detection accuracy.

Building upon the breakthroughs of CNNs, the introduction of Transformer architectures has driven a new wave of development in image segmentation. Unlike CNNs, which rely on local receptive fields to extract features, Transformers utilize the self-attention mechanism to globally capture relationships between distant pixels in an image [28]. This allows Transformers to better address the issue of long-range dependencies while providing enhanced global feature modeling capabilities. In recent years, Transformer-based image segmentation models have rapidly emerged, including Segmenter [29], SegFormer [30], and MaskFormer [31]. Zhao et al. [32] introduced a Cross-Supervision Contrastive Learning Domain Adaptation Network that combines CNNs and Transformers to integrate local and global information from different domains for comprehensive feature extraction, achieving outstanding performance in steel defect segmentation tasks under complex multi-scenario conditions. Ma et al. [33] proposed a Transformer-based Network with Feature Complementary Fusion, featuring an Information Complementary Fusion module for merging encoding branches, and a multi-dimensional attention module to optimize long-range dependencies on crack detection datasets.

As defect segmentation models continue to advance, the limitations of single models in addressing the diverse and dynamic nature of industrial defect scenarios have become increasingly evident. On the one hand, models tailored to specific tasks or scenarios often require huge volumes of labeled data for training, which is both costly and challenging to acquire. On the other hand, the generalization ability of these models in complex settings needs further improvement, especially when faced with diverse defect patterns or complicated backgrounds.

### 2.2. Segment Anything Model

In recent years, large-scale foundation models have made remarkable breakthroughs in deep learning, demonstrating outstanding generalization capabilities and immense potential for applications. With their enormous parameter scales and training on vast datasets, models such as LLaMA [12] and GPT-4 [13] have not only advanced artificial intelligence but also left a profound impact across numerous fields [14]. Building on the success of foundation models in NLP, researchers have begun exploring their potential applications in computer vision domains, such as CLIP [34] for text-to-image generation and Grounding-Dino for open-world object detection [35].

The Segment Anything Model (SAM) [20], proposed by Meta AI, is a versatile image segmentation model designed to efficiently segment any image content using human prompts (e.g., points, boxes, and text). The architecture of SAM consists of three components: the image encoder, the mask decoder, and the prompt encoder. Inspired by large models from NLP, the image encoder uses a Vision Transformer (VIT) [28] based on pre-trained MAE [36], which extracts deep image embeddings from processed images. The prompt encoder transforms human-generated prompts into high-dimensional embeddings to assist with decoding. The lightweight mask decoder decodes both the image and prompt embeddings to generate the final segmentation mask. Benefiting from its pre-training on large-scale data, SAM demonstrates exceptional generalization capabilities and flexibility, achieving excellent performance across a variety of downstream tasks [37,38,39,40]. However, despite SAM’s success in general image segmentation, its applicability in specialized domains remains limited [21]. In particular, industrial defect segmentation presents challenges due to the diverse defect morphologies, imbalanced scales, and complex background noise [22]. The pre-training of SAM is primarily based on natural image data, which lack domain-specific knowledge representation for such tasks. Furthermore, SAM requires human prompts for segmentation, which incurs high costs in industrial environments and lacks category-specific information [23], making it unsuitable for large-scale automated defect detection.

### 2.3. Adaptation of SAM

SAM has a vast number of parameters, and the large computational cost limits its practical applicability in real-world scenarios. Fine-tuning is a common method for adapting pre-trained models to specific tasks, but traditional fine-tuning approaches often require updating most of the parameters, which considerably elevates both computational and storage demands. To strike a balance between performance and parameter efficiency, Parameter-Efficient Fine-Tuning (PEFT) has been introduced into the adaptation of SAM. PEFT selectively adjusts only a subset of model parameters, reducing both computational and storage costs, while keeping the majority of parameters intact [15]. PEFT techniques include adapters [16], selective parameter adjustments [17], prompt-driven techniques [18], and Low-Rank Adaptation (LoRA) [19] within the domain of NLP. Chen et al. [41,42] improved SAM’s performance in low-resource tasks, such as camouflage object detection and shadow segmentation, by introducing lightweight adapters and using visual prompts to inject task-specific knowledge into SAM. Zhang et al. [24] leveraged LoRA to fine-tune SAM’s image encoder, as well as the prompt encoder and mask decoder, on annotated multi-organ image datasets. The mask decoder was also modified to adapt it for semantic segmentation tasks. Ye et al. [43] removed the prompt encoder and connected a LoRA-fine-tuned SAM image encoder directly to a MaskFormer decoder, redesigning the feature extractor to seamlessly integrate with the image encoder. This approach addresses a key gap in crack detection and size estimation research. Similarly, studies [44,45,46,47,48] have explored the potential of fine-tuning SAM with the adapter paradigm for applications in remote sensing, medicine, and agriculture.

Despite the significant progress made using these PEFT techniques in reducing fine-tuning parameters and improving training efficiency, they still have limitations when applied to defect segmentation tasks. Most of these methods focus on parameter efficiency, but do not adequately optimize image features essential for the task, such as edges, textures, or fine-grained regions, which results in a lack of deep modeling of local and contextual information. Zhong et al. [49] combined LoRA with lightweight convolutions, for a model named Conv-LoRA, to overcome the local prior limitations in the original ViT architecture. Standard LoRA drastically reduces the number of parameters and computational overhead by decomposing the frozen weight matrices in SAM into two trainable low-rank matrices, enabling the original large-scale parameter matrices to be approximated using considerably smaller low-rank matrices. Building upon the LoRA architecture, Conv-LoRA introduces a Mixture-of-Experts (MoE) [50] mechanism. Specifically, Conv-LoRA inserts an MoE gating network between the two low-rank matrices in LoRA, dynamically selecting experts to process specific image information at different feature scales. Each expert reconstructs image features at a chosen scale, followed by a 3×3 convolution operation, and then maps the features back to the default scale. It has shown superior performance over other PEFT methods across multiple domains. However, while MoE can select different convolution scales based on input features, it still does not fully exploit multi-scale features for the same input, and introduces more parameters than LoRA. To optimize these issues, we propose a PEFT technique that integrates LoRA with partial convolution, ensuring efficient multi-scale feature extraction while maintaining parameter efficiency.

Additionally, SAM’s promptable segmentation mechanism aids in image segmentation through the use of user-provided prompts. However, in industrial defect segmentation tasks, relying on manual prompts not only increases operational complexity but also fails to meet the demands of large-scale automated detection. Some studies [51,52,53] have attempted to address this using deep learning networks to pre-train downstream datasets and generate corresponding prompt points or boxes, which are then fed into the prompt encoder to assist in segmentation. In defect segmentation, on the other hand, targets are frequently small and local, blending into the background [54]. Simple point or box prompts are insufficient to fully capture the morphology and distribution of defect [55]. In contrast, mask prompts can provide pixel-level annotations to help SAM better understand the target’s location, shape, and size, especially in complex backgrounds or occlusion scenarios. Some studies [56,57,58] use small-scale traditional segmentation networks (e.g., U-Net) to generate low-resolution masks as prompts for the input image, but these generated masks perform poorly in capturing fine details and complex defect characteristics, particularly when dealing with high-resolution and heterogeneous industrial images with varying types or scales. As a result, the low-resolution masks often contain erroneous prompts and noise. In contrast, our devised IPEG automatically generates high-quality dense prompts directly from image embeddings, overcoming the limitations of conventional methods in fine-grained feature extraction and handling complex backgrounds.

## 3. Methodology

### 3.1. Overview of PA-SAM

**Figure 2:**
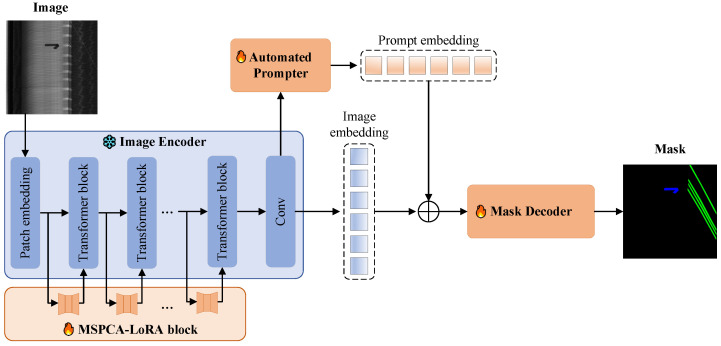
Architecture and pipeline of proposed PA-SAM for defect segmentation.

The proposed PA-SAM structure is shown in Figure 2, which builds upon SAM’s architecture. To preserve the high-quality pre-trained features of SAM, we freeze the main parameters of its image encoder (i.e., ViT) and introduce trainable MSPCA-LoRA blocks into the self-attention layers of each Transformer block. This approach avoids the need for full model fine-tuning. The defect image is first pre-processed to the input image $x\in R^{3\times 1024\times 1024}$, whose spatial resolution is 1024×1024 and channel number is 3, before entering the Image Encoder. In the image encoder, the patch embedding firstly transform the input image into continuous patch embeddings $E_{patch}\in R^{64\times 64\times 768}$. These embeddings then pass through the Transformer blocks to produce attention embeddings $E_{atten}\in R^{64\times 64\times 768}$. Each Transformer block comprises a multi-head self-attention layer and a Multilayer Perceptron (MLP) layer. The Transformer blocks are fine-tuned via the MSPCA-LoRA block with initialized parameters, while keeping the embedding dimensions unchanged. The output $E_{atten}$ from the Transformer blocks is downsampled via convolutional layers to yield the image embeddings $E_{img}\in R^{256\times 64\times 64}$. To eliminate the need for manual prompts, we devise the Image-to-Prompt Embedding Generator (IPEG). The IPEG takes the image embeddings as input, enhances multi-scale edge information, and generates prompt embeddings $E_{prompt}\in R^{256\times 64\times 64}$, step by step. The mask decoder then combines the prompt embeddings with the image embeddings to assist the decoding process. In the original SAM, the mask decoder generates segmentation masks without categorizing specific classes, so we modify the output of the decoder. The final output from the mask decoder is a predicted mask $Y\in R^{Class\text{\_}num\times 256\times 256}$, where Class_num is the number of classifications, aligned with the ground truth mask *S* via bilinear sampling. Since the mask decoder plays a crucial role in generating the final segmentation result, and is a lightweight Transformer decoder with fewer parameters compared to the image encoder, we fully fine-tune the mask decoder to optimize its performance for downstream tasks.

### 3.2. MSPCA-LoRA

The original LoRA method achieves PEFT by adapting the pre-trained model weights with low-rank matrices [19]. The core idea of LoRA is to decompose specific weight matrices in the model that require adjustment (e.g., attention weight matrices or feedforward network weight matrices in Transformers) into the product of two low-rank matrices. For an input *x*, the forward propagation process can be expressed as follows:

(1) $$y=W_{0}x+\Delta Wx=W_{0}x+W_{b}W_{a}x,$$

where the original pre-trained weight matrix $W_{0}\in R^{b\times a}$ (*a* and *b* represent the input and output dimensions, respectively) remains frozen and is not fine-tuned. The new adaptive weight adjustment $\Delta W=W_{b}W_{a}$, where $W_{b}$ and $W_{a}$ are smaller trainable low-rank matrices that satisfy the low-rank constraints $W_{b}\in R^{b\times r}$ and $W_{a}\in R^{r\times a}$, with r≪min(a,b). This method allows fine-tuning to focus solely on training the low-rank matrices $W_{b}$ and $W_{a}$, rather than the entire weight matrix, leading to a marked reduction in computational and storage costs.

The authors of [49] showed that the pre-training of SAM impedes the ability of its image encoder to learn high-level semantic information. ViT, which mainly relies on global self-attention mechanisms to process image data [28], is less effective than CNNs in capturing local features and dependencies. The lack of local priors can limit performance, especially for tasks that require fine-grained local information (e.g., semantic segmentation and defect detection). By introducing convolution layers between the low-rank matrices in LoRA, we combine the local feature-capturing ability of convolutions with the PEFT advantages of LoRA. Convolution operations inherently excel at capturing local features, which enhances ViT’s sensitivity to local details. Additionally, the size and number of convolution kernels can be controlled through low-rank constraints, preventing excessive model complexity while retaining LoRA’s efficiency. While ViT’s feature maps have consistent scales, object masks often span a wide range of scales. Therefore, multi-scale feature extraction is essential for incorporating and aggregating local prior knowledge across scales. However, multi-scale strategies, though effective in utilizing this prior knowledge, increase computational costs. To efficiently capture multi-scale local features, we propose the Multi-Scale Partial Convolution Aggregation (MSPCA) module. This module applies convolution kernels of varying sizes, extracting multi-scale features from the image layer by layer and partially.

**Figure 3:**
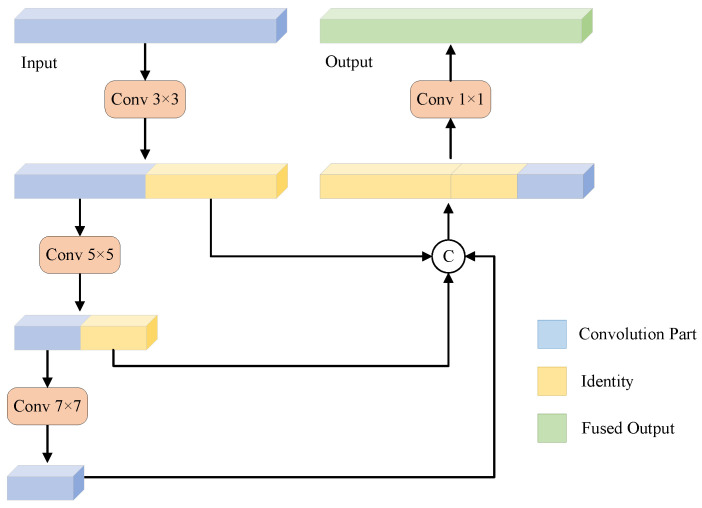
The structure of Multi-Scale Partial Convolution Aggregation (MSPCA).

The structure of the MSPCA module is illustrated in Figure 3. This module incorporates several convolution layers with varying receptive fields, using convolution kernels of 3×3,5×5, and 7×7. These layers extract image features across different scales, capturing multi-level information from local to global. To reduce the parameter count and improve computational efficiency, MSPCA applies partial convolution (PConv) [59] to hierarchically reduce channel dimensions on selected branches. PConv leverages the redundancy in feature maps by systematically applying a regular convolution to only a subset of input channels, while leaving the rest unaffected. For example, in the first layer, after performing a 3×3 convolution on the input feature maps, the feature maps are split along the channel dimension into two parts. Then, a 5×5 convolution is applied to the first half (marked in blue in Figure 3), while the second half (marked in yellow) is retained for the final feature fusion. Essentially, PConv incurs fewer FLOPs compared to regular convolution [59]. For multi-scale feature maps, PConv performs spatial feature extraction on only part of the input channels and then fuses the feature maps from different scales using a 1×1 convolution. This architecture preserves rich, multi-scale information while lowering computing costs. Given an input feature map $X\in R^{C\times H\times W}$, where *C* is the number of channels, and *H* and *W* are the height and width, respectively, the forward propagation process of MSPCA can be expressed as follows:

(2) $$\begin{array}{cc} X_{1} & ={\mathrm{Conv}}_{3\times 3}\left(X\right),\\ X_{1a},X_{1b} & =\mathrm{Chunk}(X_{1},2,\dim=1),\\ X_{2} & ={\mathrm{Conv}}_{5\times 5}\left(X_{1a}\right),\\ X_{2a},X_{2b} & =\mathrm{Chunk}(X_{2},2,\dim=1),\\ X_{3} & ={\mathrm{Conv}}_{7\times 7}\left(X_{2a}\right),\\ X_{\mathrm{concat}} & =\mathrm{Concat}(X_{3},X_{2b},X_{1b},\dim=1),\\ Y & ={\mathrm{Conv}}_{1\times 1}\left(X_{\mathrm{concat}}\right), \end{array}$$

where ${\mathrm{Conv}}_{k\times k}\left(\cdot \right)$ represents a convolution operation with a k×k kernel, Chunk(·,2,dim) splits the feature map along the channel dimension into two parts, and Concat(·,dim) concatenates multiple feature maps along the channel dimension. In the generated feature map, $X_{1}\in R^{C\times H\times W},X_{1a},X_{1b},X_{2}\in R^{\frac{C}{2}\times H\times W},X_{2a},X_{2b},X_{3}\in R^{\frac{C}{4}\times H\times W},X_{\mathrm{concat}}\in R^{(\frac{C}{4}+\frac{C}{4}+\frac{C}{2})\times H\times W},$ and $Y\in R^{C\times H\times W}$.

**Figure 4:**
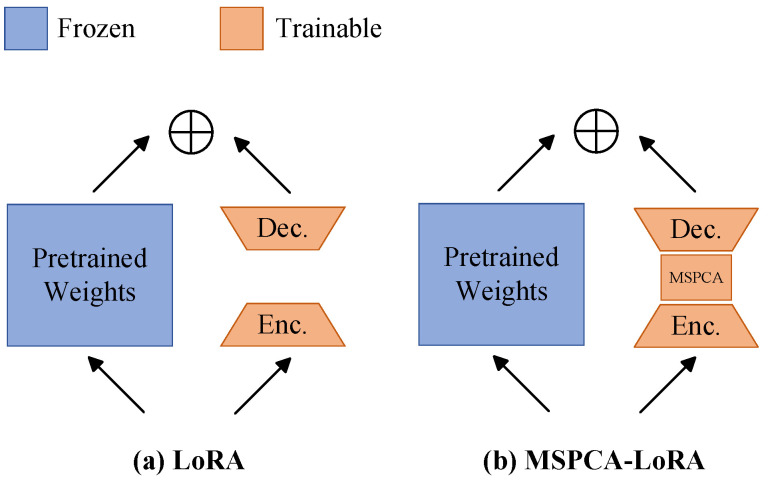
Comparison of the forward propagation process between (**a**) LoRA and (**b**) MSPCA-LoRA.

LoRA primarily utilizes global low-rank matrices, but it struggles with capturing local feature maps. To overcome this limitation, we introduce MSPCA into LoRA’s low-rank linear layer, MSPCA-LoRA, as illustrated in Figure 4. Similarly to LoRA, MSPCA-LoRA begins by freezing the pre-trained model weights. Then, it incorporates two low-rank matrices, $W_{A}$ and $W_{B}$, to perform adaptive low-rank adjustments on the input. Matrix $W_{A}$ reduces the input feature maps to a low-rank space, while matrix $W_{B}$ re-expands these feature maps back into the output space. Then, we apply the MSPCA module to the output of matrix $W_{A}$ to extract multi-scale and fine-grained local feature maps. This process not only preserves the Low-Rank Adaptation benefits but also enhances the model’s ability to capture local image structures. Matrix $W_{B}$ then transforms the feature maps processed by MSPCA. Finally, the transformed feature maps fuse with the output that comes from the main linear layer through scaling and addition. Mathematically, the forward propagation process of MSPCA-LoRA can be derived from Equation (1) as follows:

(3) $$y=W_{0}x+W_{b}\left(\mathrm{MSPCA}\left(W_{a}x\right)\right)\cdot \frac{\alpha }{r},$$

where $W_{0}\in R^{C_{\mathrm{out}}\times C_{\mathrm{in}}}$, $W_{a}\in R^{C_{\mathrm{in}}\times C_{\mathrm{in}}}$, $W_{b}\in R^{C_{\mathrm{in}}\times C_{r}}$, and $x,y\in R^{C_{r}\times H\times W}$. H/W denote the spatial resolution of the input feature maps, and $C_{\mathrm{in}}/C_{\mathrm{out}}$ are the input and output channel numbers, respectively. The input and output dimensions of MSPCA(·) are both *r*. α is a scaling factor, which was set as α=r here.

**Figure 5:**
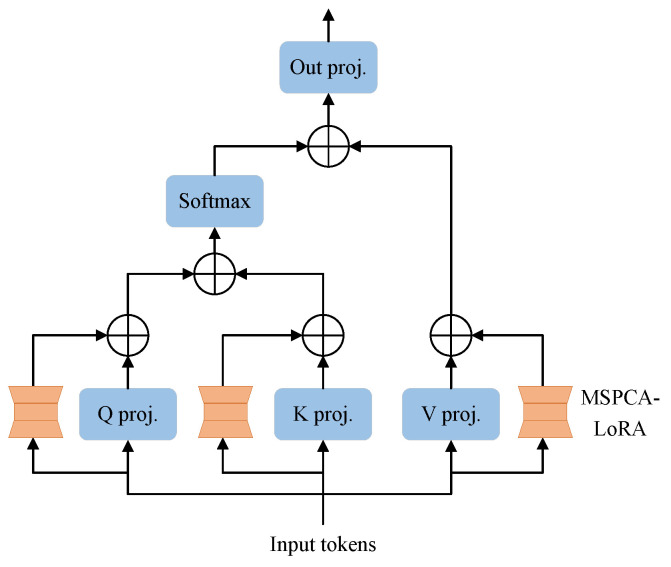
Structure of MSPCA-LoRA in Transformer block.

In the ViT architecture of the image encoder, each Transformer block contains multi-head self-attention (MHSA) and feedforward neural network (FFN) layers [28]. The MHSA layer projects the input feature maps into the attention space by means of query, key, and value projections. We introduce the MSPCA-LoRA structure into these projection layers, as illustrated in Figure 5. This approach ensures that the pre-trained model’s original information is preserved. At the same time, it efficiently adapts to the new task by leveraging LoRA and PConv to capture relevant feature maps.

### 3.3. Image-to-Prompt Embedding Generator

**Figure 6:**
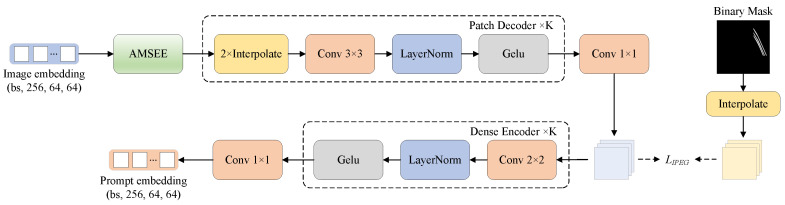
Structure of Image-to-Prompt Embedding Generator (IPEG).

To generate high-quality prompt embeddings for defect segmentation tasks, we propose an automatic prompt generator, named IPEG. This module takes image embeddings as input, and automatically generates a set of prompt embeddings to assist in segmentation decoding. Unlike directly learning prompt representations from the original image, image embeddings already encapsulate rich global semantics and local contextual information, providing a solid feature foundation for prompt generation. This, in turn, simplifies the process of learning prompt representations. The structure of the IPEG is illustrated in Figure 6. In this structure, the AMSEE enhances the edge and texture information within the input image embeddings. The Patch Decoder decodes multi-scale features from the enhanced embeddings, generating more detailed feature representations. Finally, the Dense Encoder further compresses and refines these features, producing the final high-quality prompt embeddings.

**Figure 7:**
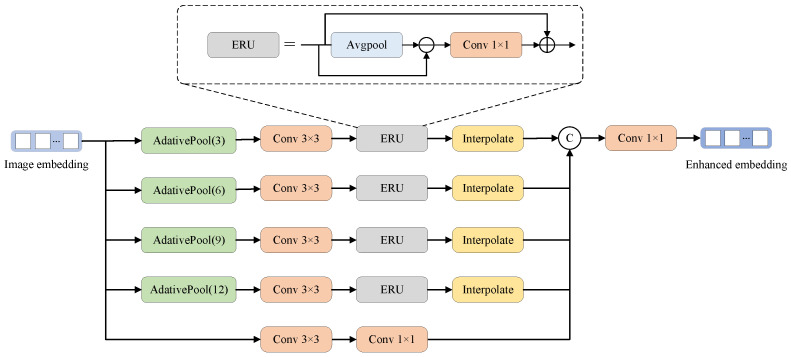
Structure of Adaptive Multi-Scale Edge Enhancer (AMSEE).

Defect regions often exhibit intricate patterns and boundary characteristics. To enhance the IPEG’s ability to detect complex defect boundaries and details, we designed the Adaptive Multi-Scale Edge Enhancer (AMSEE) module. This module further processes the high-dimensional embeddings. As shown in Figure 7, the AMSEE consists of three main components: multi-scale adaptive pooling, edge information enhancement, and feature fusion operations. First, multi-scale adaptive pooling extracts local information from the image embeddings at different scales. Next, the Edge Refinement Unit (ERU) filters out low-frequency information and extracts edge details. Specifically, the ERU first applies average pooling to the input embeddings to perform smoothing. Then, it subtracts the smoothed features from the original input embeddings. This step removes low-frequency components and emphasizes the edge features. Afterward, the enhanced edge features are processed through convolution and added back to the original input embeddings, forming the enhanced output. Once the ERU has enhanced the edge features, the feature fusion operation aligns and concatenates features from multiple scales through interpolation. Finally, a 1×1 convolution is applied to generate a unified, enhanced embedding representation. For the input image embedding $E_{img}\in R^{256\times 64\times 64}$, the forward propagation process of the AMSEE can be expressed as follows:

(4) $$\begin{array}{cc} E_{k} & =\mathrm{AdaptivePool}\left(E_{img},k\right),k\in \left\{3,6,9,12\right\},\\ E_{k}^{\prime } & ={\mathrm{Conv}}_{3\times 3}\left(E_{k}\right),\\ E_{k}^{\prime \prime } & =\mathrm{ERU}(E_{k}^{\prime }),\\ {\tilde{E}}_{k} & =\mathrm{Interpolate}(E_{k}^{\prime \prime },256,256),\\ E_{base} & ={\mathrm{Conv}}_{1\times 1}\left({\mathrm{Conv}}_{3\times 3}\left(E_{img}\right)\right),\\ E_{img}^{\prime } & ={\mathrm{Conv}}_{1\times 1}\left(\mathrm{Concat}({\tilde{E}}_{3},{\tilde{E}}_{6},{\tilde{E}}_{9},{\tilde{E}}_{12},E_{\mathrm{base}})\right), \end{array}$$

where k∈{3,6,9,12} are the scale sizes for multi-scale adaptive pooling, $E_{k}$ is the pooled embedding at scale *k*, $E_{k}^{\prime }$ is the embedding after convolution is applied to the pooled embedding, $E_{k}^{\prime \prime }$ is the edge-enhanced embedding obtained with the ERU, ${\tilde{E}}_{k}$ is the edge-enhanced embedding aligned to the original resolution of $E_{img}$ via interpolation, $E_{base}$ is the base embedding representation serving as the reference for the enhancement process to retain essential global information, and $E_{img}^{\prime }\in R^{256\times 64\times 64}$ is the final fused embedding.

The AMSEE processes the image embeddings $E_{img}$ to produce the enhanced embeddings $E_{img}^{\prime }$, which are then fed into the Patch Decoder to generate the auxiliary mask. The Patch Decoder consists of *K* layers, each containing an upsampling layer, a 3×3 convolution layer, LayerNorm, and GELU activation. It gradually reduces the number of channels in the embeddings while increasing its spatial resolution, generating more detailed feature representations. In the final layer, a 1×1 convolution compresses the decoded embeddings $D_{k}$ to match the target number of channels, generating the auxiliary mask $\hat{P}$. To optimize the decoding process of the IPEG, we apply binary cross-entropy (BCE) loss to supervise the generated auxiliary mask, which is expressed as

(5) $$L_{IPEG}=-\frac{1}{N}\sum_{i=1}^{N}\left[p_{i}\log\left({\hat{p}}_{i}\right)+\left(1-p_{i}\right)\log\left(1-{\hat{p}}_{i}\right)\right],$$

where $p_{i}\in P$ represents the ground truth value of the *i*-th pixel in the auxiliary mask, *P* is the binarized and interpolated segmentation map obtained from the source domain mask *S*, which matches the size of $\hat{P}$, $p_{i}$ is the predicted probability, and *N* is the total number of pixels. This loss function guides the model to more accurately generate high-quality auxiliary masks by minimizing the difference between the ground truth and predicted values.

The generated auxiliary mask $\hat{P}$ is then passed into the Dense Encoder module to generate the prompt embeddings. The Dense Encoder consists of *K* layers, each containing a 2×2 convolution, LayerNorm, and GELU activation. Its main function is to gradually downsample the auxiliary mask $\hat{P}$, ensuring that its resolution aligns with the input image embeddings. Through this process, the Dense Encoder compresses the spatial dimensions of the embeddings while preserving multi-scale information, allowing the generated prompt embeddings to align with the dimensions of the image embeddings.

### 3.4. Multi-Class Mask Decoder

**Figure 8:**
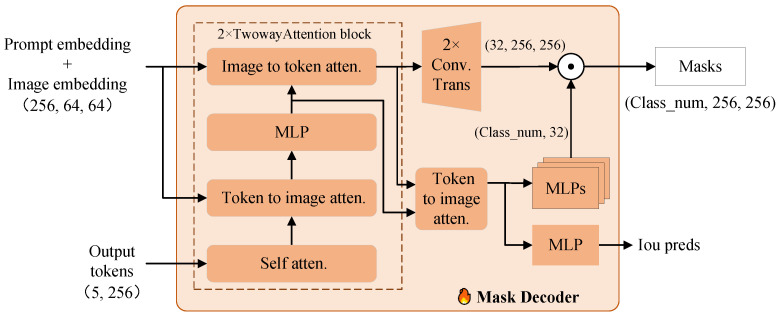
Structure of multi-class mask decoder.

The mask decoder can be regarded as a combination of a lightweight Transformer decoder and an image segmentation head, as illustrated in Figure 8. It takes image embeddings and prompt embeddings as mixed embeddings $E_{mix}\in R^{256\times 64\times 64}$, along with learnable output tokens $T_{out}\in R^{5\times 256}$. The output tokens are similar to the classification (cls) tokens in ViT, consisting of two learnable tokens: IoU tokens $T_{iou}\in R^{1\times 256}$ and mask tokens $T_{mask}\in R^{4\times 256}$, which are concatenated to assist in generating predicted masks. The input embeddings are updated iteratively using the cross-attention mechanism within the Twowayattention module, which facilitates the interaction between image embeddings and tokens. Twowayattention operates in four stages: (1) self-attention applied to tokens; (2) cross-attention from tokens to embeddings; (3) pointwise MLP operations on tokens; (4) cross-attention from embeddings to tokens. The embeddings resulting from step (4) are processed via a deconvolution operation to generate upsampled embeddings $E_{up}\in R^{32\times 256\times 256}$. Subsequently, the embeddings from steps (2) and (4) undergo a second round of cross-attention, from tokens to embeddings, and are processed via hypernetwork-based parallel MLP operations (MLPs) to yield hyper features $Hyper_{in}$. In the original SAM, the output dimension of the MLPs is set to 4. The mask decoder then employs a boolean parameter to determine whether to generate multi-scale binary masks for the same prompt, i.e., ambiguous segmentation [24]. To make SAM suitable for multi-class semantic tasks, we modified the output dimension of the MLPs based on the number of classes *C*. Finally, $Hyper_{in}$ undertakes a dot product operation with $E_{up}$ to produce the predicted mask $M\in R^{C\times 256\times 256}$. The modified multi-class mask decoder is able to output the corresponding number of maps for specified tasks, each representing the segmentation prediction for the same class region, including the background.

For a predicted mask $X\in R^{C\times H\times W}$ obtained by interpolating and aligning M, our goal is to predict a segmentation map $\hat{S}$ of resolution H×W. Each pixel in $\hat{S}$ belongs to a class from the predefined class list $Y=\left\{y_{0},y_{1},\ldots ,y_{K}\right\}$, and is as close as possible to the ground truth *S*, where $y_{0}$ represents the background class, and $y_{1}$ to $y_{k}$ represent different defect categories. In the inference process, the predicted map $\hat{S}$ is input into the softmax function to obtain the class probability distribution for each category:

(6) $$\hat{S}\text{\_}probs=\mathrm{Softmax}\left(\hat{S},\dim=1\right).$$

Then, for each pixel (h,w), the class with the highest probability is selected as the predicted class for the pixel:

(7) $$\hat{S}\left(h,w\right)=\arg\max_{c}\left(\hat{S}\text{\_}probs\left[c,h,w\right]\right),\;\mathrm{for}\;\mathrm{each}\;\mathrm{pixel}\;\left(h,w\right).$$

The final segmentation map $\hat{S}$ is a predicted map of size H×W, where each pixel (h×w) indicates the class that it belongs to. The objective is to most accurately reconstruct the class information from the ground truth *S*.

### 3.5. Loss Function for Segmentation

The defect regions (i.e., positive class) are typically much smaller than background regions (i.e., negative class), which leads the model to become biased towards predicting the background class, often neglecting or misclassifying defect areas. To address this issue, we employ a combination of Focal Loss and Dice Loss to supervise the predicted masks generated by the mask decoder:

(8) $$L_{Seg}=\lambda_{1}L_{Dice}+\lambda_{2}L_{Focal},$$

where $\lambda_{1}$ and $\lambda_{2}$ are weighting parameters that balance the contributions of each loss.

Dice Loss is a loss function specifically designed to handle class imbalance in segmentation tasks. It calculates the overlap between the predicted segmentation and the ground truth labels, providing more precise optimization, particularly when the target domain is small. The formula for Dice Loss is given by

(9) $$L_{Dice}=1-\frac{2\sum_{c=0}^{C-1}\sum_{i=1}^{N}p_{i}^{c}g_{i}^{c}}{\sum_{c=0}^{C-1}\sum_{i=1}^{N}p_{i}^{c}+\sum_{c=0}^{C-1}\sum_{i=1}^{N}g_{i}^{c}+\epsilon },$$

where *C* is the total number of classes, and *N* is the total number of pixels in the image. $p_{i}^{c}$ is the predicted probability that the *i*-th pixel belongs to the *c*-th class, and $g_{i}^{c}$ indicates whether the *i*-th pixel in the ground truth belongs to the *c*-th class. ϵ is a small constant included to prevent division by zero.

Focal Loss adjusts the standard CE loss function by assigning higher weights to hard-to-classify samples, guiding the model to focus more on rare positive samples (e.g., defect areas). The formula for Focal Loss is given by

(10) $$L_{Focal}=-\sum_{c=0}^{C-1}\alpha_{c}\sum_{i=1}^{N}{\left(1-p_{i}^{c}\right)}^{\gamma }\log\left(p_{i}^{c}\right),$$

where *C*, *N*, and $p_{i}^{c}$ are consistent with those defined in Equation (9), $\alpha_{c}$ is a balancing factor used to adjust the loss weight across different classes, and γ is a modulation factor used to control the rate at which the loss for difficult samples decays.

## 4. Experiments and Analysis of Results

### 4.1. Datasets

To evaluate the effectiveness and applicability of our proposed PA-SAM, we conducted extensive and convincing experiments on two industrial surface defect datasets: the SD-Saliency-900 dataset and the Tire-Seg dataset.

(1) SD-saliency-900 Dataset: This dataset [60] contains a range of challenging industrial images, characterized by complex scenarios such as low contrast and variations in defect scale. Specifically, the dataset includes three defect types with 300 images for each type: patches, inclusions, and scratches. Each image has an original resolution of 200 × 200. We selected 600 images for the training set and 300 images for the test set.

(2) Tire-Seg Dataset: The Tire detection dataset [61] is a standardized database created to address the task of automatic tire defect recognition. It consists of X-ray images of tires captured by multiple X-ray machines, with annotations performed by experienced workers, covering seven different defect types. Each raw image has a resolution of 900 × 900 and is annotated with tightly fitting bounding boxes for each defect. However, since the task requires pixel-level segmentation, these bounding box annotations are not directly suitable for training SAM. Therefore, we categorized four typical defect types (cords-overlap, cords-distance, foreign-matter, and cord-separate) and performed pixel-level annotations using the publicly available LabelMe tool. This modified dataset is referred to as the Tire-Seg dataset. We selected 860 images for the training set and 377 images for the test set. Due to the complex surface structure of tires, defects from different categories often exhibit similar characteristics and have low contrast against the background. These factors extremely increase the challenges associated with tire defect segmentation.

### 4.2. Implementations

All experiments were implemented on Ubuntu 22.04 with Pytorch 2.0.0. We used a distributed training setup utilizing three Nvidia RTX 3090 GPUs (each with 24 GB of memory) with CUDA version 11.8, alongside an Intel Xeon E5 2.50 GHz CPU. We used AdamW as the optimizer, with a learning rate of 0.001, and weight decay set to 0.1. The optimizer’s first moment estimate $\beta_{1}$ and second moment estimate $\beta_{2}$ were set to 0.9 and 0.999, respectively. During the training phase, we employed a learning rate adjustment strategy combining warm-up (with the warm-up stage lasting for 10% of the total training steps and starting at a learning rate of 0.001) and polynomial decay (with a decay exponent of 0.9) to prevent gradient instability and overfitting in the early stages. The batch size and number of epochs were set to 4 and 400, respectively. The weights in the segmentation loss function were set to $\lambda_{1}=1$ and $\lambda_{2}=1$. The balancing factor for the background class in Focal Loss was set to $\alpha_{0}=0.25$, and for the foreground classes, $\alpha_{1}$∼$\alpha_{C-1}=0.75$, with the modulation factor γ=2. The constant in Dice Loss was set to $\epsilon =1\times 10^{-4}$. For both training and testing, all images were resized to 1024 × 1024 resolution. In the image encoder, we leveraged VIT-B as the pre-trained network. In MSPCA-LoRA, we set the rank r=12 for the low-rank matrix. In the IPEG, the number of Patch Decoder and prompt encoder layers was set to K=2.

### 4.3. Evaluation Metrics

To undertake a comprehensive evaluation of the segmentation results, especially when facing challenges such as class imbalance and fine-grained segmentation, we utilized the mean Intersection over Union (mIoU) and mDice as the primary metrics for our experiments. IoU is a standard metric for measuring the overlap between the predicted segmentation and the ground truth. The Dice coefficient quantifies the similarity between the predicted region and the ground truth region. Both mIoU and mDice represent the average values of these metrics across all classes.

### 4.4. Comparison Study

To validate the generalization of PA-SAM in defect segmentation tasks, we compared it with several commonly used segmentation methods, including U-Net [7], DeeplabV3+ [62], Segmenter [29], Mask2Former [63], and PIDNet [64], on each dataset, as shown in Table 1. All compared models were trained with full parameters, and the training hyperparameters were set as described in Section 4.2.

**Figure 9:**
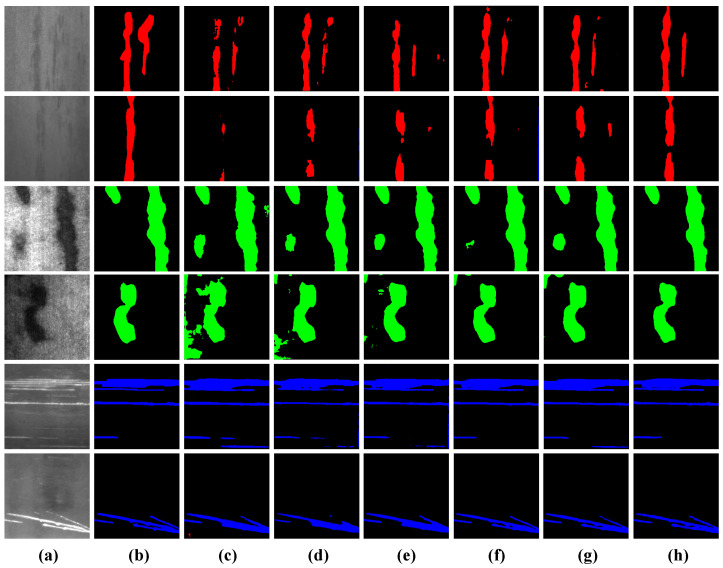
Qualitative comparison results on the SD-Saliency-900 dataset. (**a**) Image. (**b**) Ground truth. (**c**) U-Net. (**d**) Deeplabv3+. (**e**) Segformer. (**f**) Mask2former. (**g**) PIDNet. (**h**) PA-SAM.

**Figure 10:**
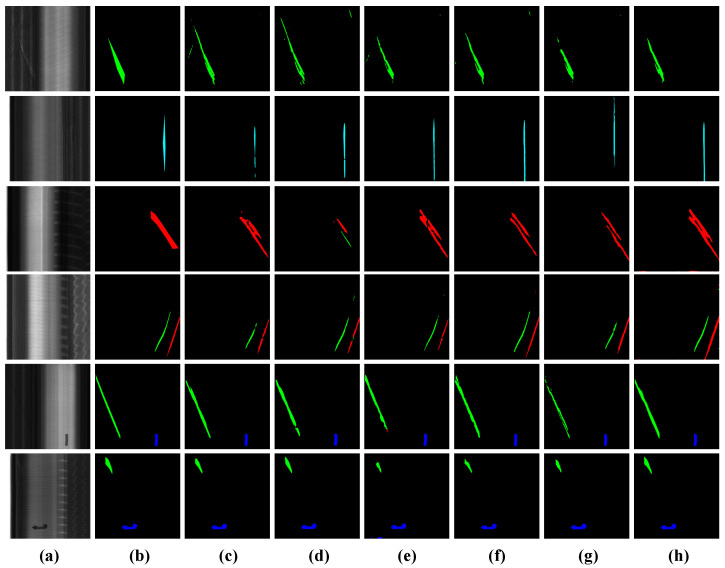
Qualitative comparison results on the Tire-Seg dataset. (**a**) Image. (**b**) Ground truth. (**c**) U-Net. (**d**) Deeplabv3+. (**e**) Segformer. (**f**) Mask2former. (**g**) PIDNet. (**h**) PA-SAM.

(1) Results on SD-saliency-900 Dataset: The results in Table 1 show that PA-SAM outperforms the other algorithms by a significant margin on evaluation metrics. Specifically, PA-SAM improves mIoU by 11.66%, 8.60%, 7.35%, 2.14%, and 2.60% over competing methods and achieves improvements of 10.45%, 6.53%, 5.40%, 1.16%, and 2.04% in mDice. These results validate that PA-SAM exhibits excellent generalization capability in typical industrial scenarios. As shown in Figure 9, we visualize the segmentation results of PA-SAM and other comparison algorithms. It can be observed that PA-SAM shows superior edge continuity and defect region localization, particularly in the presence of significant noise and low contrast in metal backgrounds. While other algorithms misclassify the background as defects (especially in rows 1–4), PA-SAM more accurately localizes defect regions, mitigating the effect of noise. In rows 5–6, PA-SAM excels in finely segmenting narrow and small defect classes, demonstrating a fine-grained ability to capture defect contours.

(2) Results on Tire-Seg Dataset: Due to the intricate surface textures of tires and the high similarity among inter-class defects, the segmentation task on this dataset is particularly challenging. Given the increased difficulty of this dataset, the overall evaluation metrics for the compared algorithms were significantly lower than those on the SD-saliency-900 experiment, with some algorithms demonstrating poor generalization performance. Despite these challenges, PA-SAM consistently outperforms other competing methods in overall performance. Specifically, compared to the other algorithms, PA-SAM achieves performance gains of 2.14%, 2.62%, 7.05%, 1.43%, and 3.58% in mIoU, and 6.43%, 9.85%, 5.50%, 1.45%, and 3.84% in mDice. On the Tire-Seg dataset, we further present the visualization results of the tire defect segmentation task, as shown in Figure 10. From the original images, it is evident that the X-ray imaging of tire defects reveals highly variable surface structures where defects frequently blend with complex and noisy backgrounds. In rows 1, 2, and 4, the defect boundaries are blurred in both the original images and ground truth, with several algorithms misclassifying the tire crown background as part of the defect. In contrast, PA-SAM segments the defect regions more accurately while maintaining edge continuity. Row 3 shows how striped background noise leads all algorithms to split the defect into two sections, while PA-SAM preserves its integrity. In rows 5–6, where high contrast between the background and defect enhances segmentation performance, PA-SAM’s results are closer to the ground truth in both defect location and morphology.

In summary of the above comparative experimental results, our proposed PA-SAM demonstrates high precision and robustness on both datasets, achieving a clear performance improvement over other methods. This result further confirms the substantial potential of SAM in industrial applications. Through knowledge transfer and automatic auxiliary decoding, PA-SAM effectively leverages the powerful generalization capability of visual foundation models for specific downstream tasks.

To assess the fine-tuning effect of MSPCA-LoRA within the SAM framework, we conducted two comparative experiments: LoRA [19] and Conv-LoRA [49]. LoRA fine-tunes using only learnable linear layers, while Conv-LoRA integrates lightweight convolution with LoRA, incorporating prior knowledge from the ViT and utilizing the MoE mechanism to dynamically select the optimal convolution scale. To ensure a fair comparison, we consistently used the default embeddings as the input to the prompt encoder, with the rank of the low-rank matrix kept constant across all experiments. In Table 2, we compare the number of parameters for each method under different low-rank constraints *r*, and Table 3 presents the experimental results for the three PEFT methods on different datasets with low-rank constraints r=12.

As shown in Table 2, MSPCA-LoRA outperforms Conv-LoRA in terms of the number of parameters. For different settings of *r*, ranging from 4 to 24, the number of parameters for all three PEFT methods increases as *r* increases. Thanks to the low-rank constraint, the addition of convolution does not significantly increase the computational cost of LoRA. Moreover, the parameter growth for MSPCA-LoRA is noticeably smaller than that for Conv-LoRA. This difference arises from the partial convolution and local channel fusion mechanism introduced in MSPCA-LoRA, which effectively reduces unnecessary parameter expansion, improving computational efficiency while maintaining model accuracy. Table 3 shows that both PEFT methods incorporating convolution outperform the pure LoRA fine-tuning method in terms of mIoU and mDice. This suggests that introducing the local prior features of VIT into LoRA significantly enhances SAM’s generalization ability. Although MSPCA-LoRA incurs lower computational costs than Conv-LoRA, it still slightly outperforms Conv-LoRA in terms of performance metrics. This result highlights that the multi-scale feature fusion mechanism, as opposed to the MoE-based fixed convolution scale selection method, is able to inject richer prior knowledge, thereby enhancing segmentation performance and robustness, particularly when dealing with fine-grained features and complex backgrounds.

### 4.5. Ablation Study

To verify the efficacy of each proposed module, we conducted a comprehensive ablation study on each dataset, as shown in Table 4. We removed the devised modules from PA-SAM sequentially and used the standard, unfine-tuned SAM as the baseline for comparison. We kept all experimental parameter settings consistent with those of PA-SAM. The two proposed primary modules (MSPCA-LoRA and IPEG) were applied independently in the experiments. To better illustrate the impact of each module on SAM, we report the number of trainable parameters (M) and their corresponding ratio (%) relative to the total number of parameters.

Firstly, the standard SAM, leveraging its vast pre-trained knowledge, still achieved reasonable accuracy metrics even when the image encoder was frozen. However, its performance was generally subpar compared to the other segmentation models discussed earlier. On the one hand, after incorporating MSPCA-LoRA, the mIoU metric improved by 22.60% and 22.97% across the two datasets, while the Dice metric increased by 20.10% and 34.99%. It is worth noting that the total trainable parameters of MSPCA, along with the two lightweight modules (mask decoder and IPEG) account for only 22.60% of the total, highlighting the crucial role of MSPCA-LoRA in enhancing the generalization ability of SAM. Furthermore, the nature of PEFT enables knowledge transfer for industrial defects at the cost of relatively modest trainable parameters. On the other hand, the IPEG module, which solely provides prompt embeddings to assist the mask decoder in segmentation, plays a less dominant role in the training process compared to the MSPCA-LoRA. Consequently, its trainable parameters were much lower than that of PEFT. Nevertheless, this lightweight auxiliary segmentation module still provides a notable improvement in metrics for SAM across both datasets. Overall, MSPCA-LoRA enhances SAM’s capacity to fuse multi-scale features and capture fine-grained information, playing a pivotal role in fine-tuning SAM. Meanwhile, the IPEG, with its lightweight design and efficient processing of image embeddings, allows SAM to more accurately locate defect areas without the need for manual prompts.

## 5. Conclusions

In this work, we proposed PA-SAM, a novel framework based on SAM for industrial defect segmentation. Specifically, we first introduced MSPCA-LoRA, a simple yet efficient PEFT method, into the image encoder to fine-tune SAM. By integrating the MSPCA into LoRA, we effectively address the issue of incomplete prior knowledge in ViTs, while preserving the efficiency of PEFT for transferring industrial defect knowledge. Additionally, the IPEG was designed to process high-dimensional embeddings from the image encoder, automatically generating prompt embeddings from global features to assist in segmentation decoding, thereby avoiding the difficulties of manual prompting in industrial settings. Finally, we made modest adjustments to the mask decoder architecture, enabling SAM to handle multi-class defect segmentation tasks. Through extensive experiments on two datasets, we show that PA-SAM conclusively outperforms other advanced algorithms in terms of segmentation performance. In summary, PA-SAM utilizes fine-tuning with partial parameters and automatic prompt generation to fully leverage the powerful generalization of SAM and modality versatility. Despite the promising performance, there still remain areas for improvement, particularly in addressing industrial defects with highly variable scales. Additionally, a lightweight adaptation of SAM is also a worthwhile direction. In the future, we aim to further investigate the potential of applying SAM to other industrial vision domains, such as 3D vision applications and anomaly detection.

## Figures and Tables

**Table 1:** Comparison results on the SD-Saliency-900 and Tire-Seg datasets.

Dataset	SD-Saliency-900		Tire-Seg	
Method	mIoU	mDice	mIoU	mDice
U-Net	62.21	74.45	59.88	73.79
Deeplabv3+	65.27	78.37	55.15	70.37
Segformer	66.52	79.50	61.25	74.72
Mask2Former	71.73	83.74	66.87	78.77
PIDNet	71.25	82.86	64.72	76.38
PA-SAM	73.87	84.90	68.30	80.22

**Table 2:** Parameter trend of three PEFT methods with varying rank *r* of LoRA.

Rank	Params (M)		
	MSPCA-LoRA	Conv-LoRA	LoRA
r=4	0.5970	0.5979	0.5967
r=8	0.6038	0.6076	0.6028
r=12	0.6108	0.6197	0.6090
r=24	0.6339	0.6695	0.6274

**Table 3:** Comparison results between MSPCA-LoRA and other PEFT methods.

Dataset	SD-Saliency-900		Tire-Seg	
PEFT Method	mIoU	mDice	mIoU	mDice
LoRA	70.70	82.29	64.08	76.43
Conv-LoRA	72.34	83.39	66.71	78.70
MSPCA-LoRA	72.76	83.70	66.87	78.91

**Table 4:** Ablation study results on the SD-Saliency-900 and Tire-Seg datasets.

Module		Params (M)/Ratio (%)	SD-Saliency-900		Tire-Seg	
MSPCA-LoRA	IPEG		mIoU	mDice	mIoU	mDice
		4.20/4.48%	50.16	63.70	43.90	53.92
✓		26.19/22.60%	72.76	83.80	66.87	78.91
	✓	5.16/5.45%	52.93	67.65	45.00	55.15
✓	✓	27.15/23.24%	73.87	84.90	68.30	80.22

## Data Availability

The original contributions presented in this study are included in the article. Further inquiries can be directed to the authors.

## References

[1] Tulbure A.A., Tulbure A.A., Dulf E.H. (2022). A review on modern defect detection models using DCNNs–Deep convolutional neural networks. J. Adv. Res..

[2] Tang B., Chen L., Sun W., Lin Z.K. (2023). Review of surface defect detection of steel products based on machine vision. IET Image Process..

[3] Ren Z., Fang F., Yan N., Wu Y. (2022). State of the art in defect detection based on machine vision. Int. J. Precis. Eng. Manuf.-Green Technol..

[4] Saberironaghi A., Ren J., El-Gindy M. (2023). Defect detection methods for industrial products using deep learning techniques: A review. Algorithms.

[5] Minaee S., Boykov Y., Porikli F., Plaza A., Kehtarnavaz N., Terzopoulos D. (2021). Image segmentation using deep learning: A survey. IEEE Trans. Pattern Anal. Mach. Intell..

[6] Shelhamer E., Long J., Darrell T. (2017). Fully convolutional networks for semantic segmentation. IEEE Trans. Pattern Anal. Mach. Intell..

[7] Ronneberger O., Fischer P., Brox T. (2015). U-net: Convolutional networks for biomedical image segmentation. Proceedings of the International Conference on Medical Image Computing and Computer-Assisted Intervention.

[8] Liang-Chieh C., Papandreou G., Kokkinos I., Murphy K., Yuille A. Semantic image segmentation with deep convolutional nets and fully connected crfs. Proceedings of the International Conference on Learning Representations.

[9] Lin T.Y., Maire M., Belongie S., Hays J., Perona P., Ramanan D., Dollár P., Zitnick C.L. (2014). Microsoft coco: Common objects in context. Proceedings of the Computer Vision—ECCV 2014: 13th European Conference.

[10] Mottaghi R., Chen X., Liu X., Cho N.G., Lee S.W., Fidler S., Urtasun R., Yuille A. The role of context for object detection and semantic segmentation in the wild. Proceedings of the IEEE Conference on Computer Vision and Pattern Recognition.

[11] Wang Y., Zhang Y., Jiang Z., Zheng L., Chen J., Lu J. (2022). Robust learning against label noise based on activation trend tracking. IEEE Trans. Instrum. Meas..

[12] Touvron H., Lavril T., Izacard G., Martinet X., Lachaux M.A., Lacroix T., Rozière B., Goyal N., Hambro E., Azhar F. (2023). Llama: Open and efficient foundation language models. arXiv.

[13] Achiam J., Adler S., Agarwal S., Ahmad L., Akkaya I., Aleman F.L., Almeida D., Altenschmidt J., Altman S., Anadkat S. (2023). Gpt-4 technical report. arXiv.

[14] Zhou C., Li Q., Li C., Yu J., Liu Y., Wang G., Zhang K., Ji C., Yan Q., He L. (2024). A comprehensive survey on pretrained foundation models: A history from bert to chatgpt. Int. J. Mach. Learn. Cybern..

[15] Han Z., Gao C., Liu J., Zhang J., Zhang S.Q. (2024). Parameter-efficient fine-tuning for large models: A comprehensive survey. arXiv.

[16] Houlsby N., Giurgiu A., Jastrzebski S., Morrone B., De Laroussilhe Q., Gesmundo A., Attariyan M., Gelly S. Parameter-efficient transfer learning for NLP. Proceedings of the International Conference on Machine Learning.

[17] Sung Y.L., Cho J., Bansal M. (2022). Lst: Ladder side-tuning for parameter and memory efficient transfer learning. Adv. Neural Inf. Process. Syst..

[18] Li X.L., Liang P. (2021). Prefix-tuning: Optimizing continuous prompts for generation. arXiv.

[19] Hu E.J., Shen Y., Wallis P., Allen-Zhu Z., Li Y., Wang S., Wang L., Chen W. (2021). Lora: Low-rank adaptation of large language models. arXiv.

[20] Kirillov A., Mintun E., Ravi N., Mao H., Rolland C., Gustafson L., Xiao T., Whitehead S., Berg A.C., Lo W.Y. Segment anything. Proceedings of the IEEE/CVF International Conference on Computer Vision.

[21] Ji W., Li J., Bi Q., Liu T., Li W., Cheng L. (2024). Segment anything is not always perfect: An investigation of sam on different real-world applications. Mach. Intell. Res..

[22] Hu B., Gao B., Tan C., Wu T., Li S.Z. (2023). Segment anything in defect detection. arXiv.

[23] Chen K., Liu C., Chen H., Zhang H., Li W., Zou Z., Shi Z. (2024). RSPrompter: Learning to prompt for remote sensing instance segmentation based on visual foundation model. IEEE Trans. Geosci. Remote Sens..

[24] Zhang K., Liu D. (2023). Customized segment anything model for medical image segmentation. arXiv.

[25] Badrinarayanan V., Kendall A., Cipolla R. (2017). Segnet: A deep convolutional encoder-decoder architecture for image segmentation. IEEE Trans. Pattern Anal. Mach. Intell..

[26] Yang B., Liu Z., Duan G., Tan J. (2024). Residual shape adaptive dense-nested Unet: Redesign the long lateral skip connections for metal surface tiny defect inspection. Pattern Recognit..

[27] Kong D., Hu X., Gong Z., Zhang D. (2024). Segmentation of void defects in X-ray images of chip solder joints based on PCB-DeepLabV3 algorithm. Sci. Rep..

[28] DosoViTskiy A., Beyer L., Kolesnikov A., Weissenborn D., Zhai X., Unterthiner T., Dehghani M., Minderer M., Heigold G., Gelly S. (2020). An Image is Worth 16 × 16 Words: Transformers for Image Recognition at Scale. arXiv.

[29] Strudel R., Garcia R., Laptev I., Schmid C. Segmenter: Transformer for semantic segmentation. Proceedings of the IEEE/CVF International Conference on Computer Vision.

[30] Xie E., Wang W., Yu Z., Anandkumar A., Alvarez J.M., Luo P. (2021). SegFormer: Simple and Efficient Design for Semantic Segmentation with Transformers. arXiv.

[31] Cheng B., Schwing A., Kirillov A. (2021). Per-pixel classification is not all you need for semantic segmentation. Adv. Neural Inf. Process. Syst..

[32] Zhao L., Zhang Y., Duan J., Yu J. (2025). Cross-supervised contrastive learning domain adaptation network for steel defect segmentation. Adv. Eng. Inform..

[33] Ma M., Yang L., Liu Y., Yu H. (2024). A transformer-based network with feature complementary fusion for crack defect detection. IEEE Trans. Intell. Transp. Syst..

[34] Radford A., Kim J.W., Hallacy C., Ramesh A., Goh G., Agarwal S., Sastry G., Askell A., Mishkin P., Clark J. Learning transferable visual models from natural language supervision. Proceedings of the International Conference on Machine Learning.

[35] Liu S., Zeng Z., Ren T., Li F., Zhang H., Yang J., Jiang Q., Li C., Yang J., Su H. (2024). Grounding dino: Marrying dino with grounded pre-training for open-set object detection. Proceedings of the European Conference on Computer Vision.

[36] He K., Chen X., Xie S., Li Y., Dollár P., Girshick R. Masked autoencoders are scalable vision learners. Proceedings of the IEEE/CVF Conference on Computer Vision and Pattern Recognition.

[37] Zhang C., Liu L., Cui Y., Huang G., Lin W., Yang Y., Hu Y. (2023). A comprehensive survey on segment anything model for vision and beyond. arXiv.

[38] Ma J., He Y., Li F., Han L., You C., Wang B. (2024). Segment anything in medical images. Nat. Commun..

[39] Cen J., Zhou Z., Fang J., Shen W., Xie L., Jiang D., Zhang X., Tian Q. (2023). Segment anything in 3d with nerfs. Adv. Neural Inf. Process. Syst..

[40] Zhang R., Jiang Z., Guo Z., Yan S., Pan J., Ma X., Dong H., Gao P., Li H. (2023). Personalize segment anything model with one shot. arXiv.

[41] Chen T., Zhu L., Deng C., Cao R., Wang Y., Zhang S., Li Z., Sun L., Zang Y., Mao P. Sam-adapter: Adapting segment anything in underperformed scenes. Proceedings of the IEEE/CVF International Conference on Computer Vision.

[42] Chen T., Lu A., Zhu L., Ding C., Yu C., Ji D., Li Z., Sun L., Mao P., Zang Y. (2024). Sam2-adapter: Evaluating & adapting segment anything 2 in downstream tasks: Camouflage, shadow, medical image segmentation, and more. arXiv.

[43] Ye Z., Lovell L., Faramarzi A., Ninić J. (2024). Sam-based instance segmentation models for the automation of structural damage detection. Adv. Eng. Inform..

[44] Pu X., Jia H., Zheng L., Wang F., Xu F. (2025). Classwise-sam-adapter: Parameter efficient fine-tuning adapts segment anything to sar domain for semantic segmentation. IEEE J. Sel. Top. Appl. Earth Obs. Remote Sens..

[45] Yan Z., Li J., Li X., Zhou R., Zhang W., Feng Y., Diao W., Fu K., Sun X. (2023). RingMo-SAM: A foundation model for segment anything in multimodal remote-sensing images. IEEE Trans. Geosci. Remote Sens..

[46] Qiu Z., Hu Y., Li H., Liu J. (2023). Learnable ophthalmology sam. arXiv.

[47] Wang A., Islam M., Xu M., Zhang Y., Ren H. (2023). Sam meets robotic surgery: An empirical study on generalization, robustness and adaptation. Proceedings of the International Conference on Medical Image Computing and Computer-Assisted Intervention.

[48] Li Y., Wang D., Yuan C., Li H., Hu J. (2023). Enhancing agricultural image segmentation with an agricultural segment anything model adapter. Sensors.

[49] Zhong Z., Tang Z., He T., Fang H., Yuan C. (2024). Convolution meets lora: Parameter efficient finetuning for segment anything model. arXiv.

[50] Shazeer N., Mirhoseini A., Maziarz K., Davis A., Le Q., Hinton G., Dean J. (2017). Outrageously large neural networks: The sparsely-gated mixture-of-experts layer. arXiv.

[51] Cao S., Wu Q., Ma L. TongueSAM: An Universal Tongue Segmentation Model Based on SAM with Zero-Shot. Proceedings of the 2023 IEEE International Conference on Bioinformatics and Biomedicine (BIBM).

[52] Seyoum Wahd A., Felfeliyan B., Zhou Y., Ghosh S., McArthur A., Zhang J., Jaremko J.L., Hareendranathan A. (2024). Sam2Rad: A Segmentation Model for Medical Images with Learnable Prompts. arXiv.

[53] Liu N., Xu X., Su Y., Zhang H., Li H.C. (2025). PointSAM: Pointly-Supervised Segment Anything Model for Remote Sensing Images. IEEE Trans. Geosci. Remote Sens..

[54] Tabernik D., Šela S., Skvarč J., Skočaj D. (2020). Segmentation-based deep-learning approach for surface-defect detection. J. Intell. Manuf..

[55] Huang Y., Yang X., Liu L., Zhou H., Chang A., Zhou X., Chen R., Yu J., Chen J., Chen C. (2024). Segment anything model for medical images?. Med. Image Anal..

[56] Shaharabany T., Dahan A., Giryes R., Wolf L. (2023). Autosam: Adapting sam to medical images by overloading the prompt encoder. arXiv.

[57] Zhang X., Liu Y., Lin Y., Liao Q., Li Y. Uv-sam: Adapting segment anything model for urban village identification. Proceedings of the AAAI Conference on Artificial Intelligence.

[58] Delle Castelle C., Spampinato F., Proietto Salanitri F., Bellitto G., Spampinato C. (2024). Leveraging SAM and Learnable Prompts for Pancreatic MRI Segmentation. Proceedings of the International Workshop on Personalized Incremental Learning in Medicine.

[59] Chen J., Kao S.h., He H., Zhuo W., Wen S., Lee C.H., Chan S.H.G. Run, don’t walk: Chasing higher FLOPS for faster neural networks. Proceedings of the IEEE/CVF Conference on Computer Vision and Pattern Recognition.

[60] Song G., Song K., Yan Y. (2020). Saliency detection for strip steel surface defects using multiple constraints and improved texture features. Opt. Lasers Eng..

[61] Wang Y., Zhang Y., Jiang Z., Zheng L., Chen J., Lu J. (2023). Prototype-based supervised contrastive learning method for noisy label correction in tire defect detection. IEEE Sens. J..

[62] Chen L.C., Zhu Y., Papandreou G., Schroff F., Adam H. Encoder-decoder with atrous separable convolution for semantic image segmentation. Proceedings of the European Conference on Computer Vision (ECCV).

[63] Cheng B., Misra I., Schwing A.G., Kirillov A., Girdhar R. Masked-attention mask transformer for universal image segmentation. Proceedings of the IEEE/CVF Conference on Computer Vision and Pattern Recognition.

[64] Xu J., Xiong Z., Bhattacharyya S.P. PIDNet: A real-time semantic segmentation network inspired by PID controllers. Proceedings of the IEEE/CVF Conference on Computer Vision and Pattern Recognition.

